# Posterior Urethral Valves and Fertility: Insight on Paternity Rates and Seminal Parameters

**DOI:** 10.3390/diseases13010021

**Published:** 2025-01-17

**Authors:** Marcello Della Corte, Simona Gerocarni Nappo, Antonio Aversa, Sandro La Vignera, Francesco Porpiglia, Cristian Fiori, Nicola Mondaini

**Affiliations:** 1 Division of Urology, Department of Oncology, School of Medicine, San Luigi Gonzaga Hospital, University of Turin, Regione Gonzole 10, 10043 Orbassano, Italy; 2 Division of Pediatric Urology, Regina Margherita Hospital, 10043 Turin, Italy; 3 Department of Experimental and Clinical Medicine, Magna Graecia University of Catanzaro, 88100 Catanzaro, Italy; 4 Department of Clinical and Experimental Medicine, University of Catania, 95123 Catania, Italy; 5 Department of Urology, Magna Graecia University of Catanzaro, Viale Europa, 88100 Catanzaro, Italy

**Keywords:** posterior urethral valves, fertility, paternity, lower urinary tract obstruction, megacystis, renal dysplasia, upper urinary tract dilation, prenatal hydronephrosis, urethral obstruction, retrograde ejaculation

## Abstract

Background: Posterior urethral valves (PUVs) represent the most common cause of male congenital lower urinary tract obstruction, often responsible for renal dysplasia and chronic renal failure. Despite recent improvements in patients’ outcomes thanks to prenatal ultrasound early diagnosis, PUVs can still impact sexual function and fertility. This study aims to review the available evidence on fertility in PUV patients, examining paternity rates and semen parameters. Methods: A review was conducted of the PubMed, Cochrane, Scopus, and Embase databases. Studies focusing on fertility and paternity outcomes in PUV patients were selected, including case reports, case series, and retrospective and prospective studies. Results: A total of 15 studies met the inclusion criteria. The review revealed that PUV patients often exhibit compromised semen parameters, including low sperm count, reduced motility, and abnormal morphology, as well as alterations in seminal plasma. PUV diagnoses are common in adults exhibiting infertility and ejaculation disorders, suggesting PUVs cannot be considered only a pediatric disease. Paternity rates among PUV patients were rarely reported in extenso, hampering the correct assessment of the overall medium paternity rate and its comparison with that of healthy individuals. Lastly, seminal parameters were assessed in a minimal cohort of patients, therefore, they could not be considered representative. Conclusions: Fertility and seminal parameters in PUV patients represent an under-investigated area. PUVs can variably and non-univocally affect fatherhood, and they may be associated with compromised semen quality. Early intervention and long-term follow-up are essential to address potential fertility issues. Future research should focus on developing targeted strategies to preserve and enhance fertility in this patient population.

## 1. Introduction

Posterior urethral valves (PUVs) are membranous structures located laterally to the veru montanum, between the seminal colliculi and the striated urethral sphincter [1], typically diagnosed during prenatal development in utero. PUVs are responsible for varying degrees of male obstructive uropathy detectable by prenatal ultrasonography (US), namely, dilation of the posterior urethra, megacystis (sometimes pseudodiverticular), oligohydramnios, ureteropyelocalicectasia, and bilateral hydronephrosis. Early urinary drainage after birth, followed by endoscopic valve resection (EVR), represents the gold standard for preserving residual renal function [2].

Nevertheless, PUVs still remain the most common cause of congenital urinary obstruction of the inferior urinary tract, leading to renal dysplasia and chronic renal failure (CRF).

The introduction of prenatal US has consistently improved the prognosis for PUV patients, allowing for timely care by pediatric specialists, thus improving clinical outcomes, and increasing the number of infants surviving up to adulthood. The last goal represents a crucial overturning of the past clinical history, when the PUV diagnosis was often made after the child presented severe complications, such as urosepsis, typically during earlier years of life [3]. However, early diagnosis does not exempt patients from continuing their pathway of care, in consideration of the PUV-related persistence of consequent chronic conditions, namely, lower urinary tract symptoms (LUTS), urinary tract infections (UTIs), upper urinary tract dilation, erectile dysfunction (ED), or infertility (IF). These patients are typically managed by adult urologists with little or no clinical experience in PUV management. In particular, despite the management principles of PUVs resembling those of adult urology, their application in adolescents and young adults assumes complex contours. In fact, while a clear model exists for the continuous care of conditions with an adult equivalent, such as asthma or diabetes, this concept cannot be extended to urological conditions like bladder exstrophy or, specifically, PUVs, which lack a true equivalent. Children and their families are accustomed to the holistic care received in a pediatric setting, which diminishes during the transition to adult care [4], often reducing the PUV patient to a mere recipient of a standardized care plan, centered on renal function preservation. Consequently, the urologists’ role is confined to the functional management of the lower urinary tract in order to preserve the upper one. The transition of the holistic approach from pediatrics into adulthood would allow the assessment of several additional aspects of the long-term outcomes for PUV patients, including psychological development, quality of life, and life goals [3].

In this scenario, the impact of PUVs on sexual function and fertility has always drawn attention due to their anatomical location, surgical treatment, and the concurrent effects that PUVs exert on the posterior urethra and bladder neck [5]. Firstly, CRF contributes to a low sperm fertility index, which may improve after kidney transplantation. Additionally, the anatomical abnormalities of the prostatic urethra, such as its abnormal dilation or the residual PUV flaps, reduce the anterograde propulsion of sperm and predispose to retrograde ejaculation (RE). Furthermore, PUVs and their residual flaps post-treatment can obstruct urinary drainage through obstructive mechanisms, promoting urine migration into the spermatic tract and causing UTIs and subsequent chronic inflammation of the testes. Lastly, PUVs can coexist in syndromic contexts, including cryptorchidism or genetic and chromosomal disorders, further aggravating sperm abnormalities [2,4,5,6,7].

In certain cases, the compensatory hypertrophy of the detrusor muscle and the anatomical or functional changes induced by the chronic obstruction can lead to bladder-neck dysfunction. Although the bladder neck is not primarily involved in PUVs, its function may be significantly impacted by secondary alterations. Bladder neck incision (BNI) has therefore been proposed to treat the most severe cases of complex urinary obstruction [8]. This technique, unlike EVR, is generally more effective for severe obstructions but carries a higher risk of retrograde ejaculation, potentially impairing fertility. Conversely, EVR is less invasive and often sufficient for localized obstructions, with a lower impact on ejaculatory function but a potentially higher risk of incomplete obstruction resolution. The choice between these approaches depends on the severity of the obstruction and, in adult patients, on the patients’ priorities and fertility-related considerations [9,10].

In occasional reports, newly diagnosed PUVs in adult patients have been described following the manifestation of IF and ejaculatory disorders. However, it should be emphasized that such cases are extremely rare and deviate from the typical presentation of PUVs in newborns and infants.

Some single-center studies conducted on patients with PUVs, diagnosed either in childhood or adulthood, have reported erectile function, ejaculatory function, and paternity rates comparable to those of the general population, as summarized in a literature review published over ten years ago [2]. However, data on seminal parameters are scarce [1,11,12].

This study aims to gather current evidence from the literature regarding fertility in patients with a history of PUVs, analyzing both paternity rates and seminal parameters, in order to highlight any recurring clinical issues, examine potential shortcomings in evaluation methods, and propose possible future models for management, follow-up, and specific care pathways.

## 2. Materials and Methods

A narrative literature review was conducted on PubMed, Cochrane, Scopus, and Embase databases to select studies on fertility and paternity in PUV patients. The search was carried out in April 2024 according to the framework PEO [13]:

- P = Population: the population under examination. Adult males were considered without further restrictions;
- E = Exposure. PUVs were equated as exposure to the genitourinary system, regardless of whether they have been treated or not, and of the type of treatment received;
- O = Outcome. As outcome measures, any parameter described in the literature was considered (conception and/or seminal parameters).

The quality assessment of the review was reported according to the Scale for the Assessment of Narrative Review Articles (SANRA Scale)by three Authors (F.P., C.F., and N.M.) [14].

The search was conducted using the terms: ‘posterior urethral valves’, ‘fertility’, ‘ejaculation’, ‘sexual dysfunction’, ‘potency’, ‘pregnancy rate’, ‘sexual function’, and ‘paternity’, combined with Boolean operators ‘AND’ and ‘OR’: (posterior urethral valve) AND ((fertility) OR (ejaculation) OR (sexual dysfunction) OR (potency) OR (pregnancy rate) OR (sexual function) OR (paternity)). The search is summarized in Table 1.

The search strategy included case reports, case series, retrospective studies, and prospective studies, as well as those not published in English, which were translated thanks to the automatic translator ChatGPT (Open AI, San Francisco, CA, USA) by querying the bot with the formula “translate this text into English [text]”. Non-original investigations (editorials, commentaries, abstracts, reviews, and book chapters) were excluded. No time restrictions were applied. The bibliographic references of the selected works were then evaluated for potential snowball sampling or citation chaining [15]. A single author (M.D.C.) conducted the literature search.

The methodological quality assessment of the included case reports and case series was performed using the tool proposed by Murad et al. by two authors (M.D.C. and S.G.N.). As an outcome measure (question 3), the report of at least one parameter between conception or semen analysis (SA) was considered. In this study, a slight modification was made to Murad et al.’s tool, excluding questions related to the ‘causality’ domain, for the following reasons: questions 4, 5, and 6 were excluded as they are more specific to adverse drug events, as suggested by the authors; and question 7 was excluded since the PEO criteria directly selected the adult population, which by definition meets the temporal criteria to allow for the assessment of fertility outcomes [16].

Descriptive statistical analysis was conducted using Microsoft^®^ Excel^®^ for Microsoft 365 MSO (Version 2404 Build 6.0.17531.20140). Absolute numbers are reported as dimensionless values, mean ± standard deviation, and percentages.

To ensure a thorough understanding of the concepts presented in this manuscript, a glossary of key terms related to male fertility and seminal parameters is provided in Table 2, according to the World Health Organization (WHO) [17].

## 3. Results

Fifteen studies meeting the inclusion criteria were evaluated, consisting of seven case reports and eight case series (Table 3). The snowballing method, applied to all the selected studies, did not identify any additional useful work. To read the work of Hama et al. [18] published in Japanese, the ChatGPT automatic translation tool was used.

The study quality assessment tool [16] indicated that the selection criteria domain (question 1), reporting (question 8), and exposure verification (question 2) were adequately addressed by the included studies. However, weaknesses included outcome ascertainment in the verification domain (question 3) in 4 studies (1 case report and 3 case series) (Table 4). Both Authors (M.D.C. and S.G.N.) who assessed the study quality assigned the same score to all evaluated works.

For the analysis, the included studies were separately evaluated as case reports and case series.

The quality of the review, assessed according to the SANRA Scale [14], scored 12 out of 12, with 2 points assigned for each item.

### 3.1. Case Reports

The seven included case reports [1,11,12,18,19,20,21] described the clinical history of seven adults, aged between 20 and 45 years (mean 32 ± 8.81). The case examined by Waligora [19] was not included in the age calculation as the full text was not retrievable; therefore, only the abstract was considered. Specifically, all the seven described individuals, who did not ever receive a diagnosis of PUV, were observed by the clinician for ejaculatory disorders, ranging from hypospermia (HYS) to anejaculation (AE), additionally five of them reported IF.

One single patient collected a small sample of semen, showing azoospermia, which was confirmed thanks to post-ejaculatory urine (PEU) analysis [1]. In two patients PEU found normal morphology with poor motility [20,21]. In the case of the remaining four patients, in three instances, baseline SA was not performed [11,18,19], and in one case, the patient could not collect the sample [12].

All the patients underwent EVR and, in two cases of glandular inflammation, antibiotic therapy was administered. One patient with positive sperm culture for *Escherichia coli*, also received deferred bladder diverticulectomy to remove the cause of bacterial reservoir, consisting of intra-diverticular post-voiding residual (PVR) [20]. In five cases, the treatment resulted in conception [1,11,12,19,20]. Among the remaining two studies, this outcome is not reported [18,21].

In four studies, post-treatment seminal parameters were reported as ‘normal’ [1,12,20,21]. One case exhibited persistent alterations of seminal plasma, such as citric acid 122 mg/mL (NV > 400) and zinc 31 pg/mL (NV 238 ± 89) [20].

In the case of Hama et al., the restoration of antegrade ejaculation was considered the sole outcome [18]. A detailed summary of the analyzed case reports is presented in Table 5.

### 3.2. Case Series

The eight analyzed case series consisted of single-center studies: seven of them were cross-sectional [6,7,8,9,10,22,23,24], and one was a retrospective study [25] (Table 4).

Overall, the initial number of PUV patients from each center ranged from 27 to 541 (mean 163.75 ± 85.71). Among these, the 346 patients who were traceable or contactable (e.g., those with available contacts and/or still alive) represented 3.88% to 97% (mean 48.8% ± 36.9%) of the PUV cases at each center. The 204 patients finally evaluated for the fertility or conception rate (CR) ranged from 3.32% to 70% (mean 33.12% ± 24.51%) of the initially enrolled cases. Specifically, out of 1210 PUV patients, 346 traceable or recruitable ones accounted for 26.29%. The 204 patients assessed for fertility represented 58.96% of those enrollable and 16.86% of the total. The evaluated groups of patients aged 16 to 57 years, with mean values ranging from 19.6 to 38 years.

Four studies described the applied surgical intervention [6,9,22,23]: EVR, BNI, or both, or perineal urethrotomy. One work did not provide complete case details [8], and three did not mention the surgical procedure [7,24,25].

In all case series, ejaculation disorders were reported, ranging from oligospermia between 5% and 38%, to AE in 4.7% to 5.97% of cases [6,7].

The fertility assessment was conducted as follows:

- In five studies, both an interview on sexual function (including CR) and an evaluation of seminal parameters were performed [7,9,22,23,24];
- In two studies, only patient interviews were conducted [2,8];
- In the remaining study, a retrospective analysis of clinical data was conducted, which included SA only in some cases [25].

### 3.3. Conception Rate

Only one work comprehensively assessed CR, reporting 11 cases of paternity out of 13 non-uremic patients, with the remaining two out of 13 not attempting to conceive, resulting in 100%. The same study noted the absence of offspring in six uremic patients [8]. More generally, Taskinen et al. described paternity rates similar to those of the general population [6]. Four studies reported varying conception percentages ranging from 7% to 49.25% [6,7,24,25]: three reported IF rates as 6.25% [25], 12% [6], and 14.2% [7], although not specifying the subjects desiring offspring or actively seeking pregnancy. One study did not involve patients attempting conception [22]. Another did not report any pregnancies achieved, not specifying if the subjects desired offspring or had made attempts [23]. The last work did not evaluate CR at all [9]. The reviewed case series are summarized in Table 6.

### 3.4. Seminal Parameters

Six studies reported seminal parameters [7,9,22,23,24,25]. Patients undergoing SA ranged from 7.7% to 100% of the enrolled subjects (mean 48.92% ± 33.94). Overall, 52 SA were described, corresponding to 0.9–53.3% of each center’s cases (average 13% ± 13.48), 25.49% of all enrolled patients, and 4.29% of patients with a mentioned PUV history.

Woodhouse et al. [7] examined nine samples, all showing normal parameters. However, in five of them (56%), increased viscosity and elevated pH (>8.0) were observed, a key sign of prostatitis due to the reduced secretion of prostatic citric acid and acid phosphatase [7,22], despite the corresponding patients not exhibiting clinical signs of prostatitis. No significant differences were found in semen volume or seminal urine concentration between individuals with slow and normal ejaculation. Almost all samples exhibited sperm cells with shaking movements, suggesting the presence of antibodies, although all the performed agglutination tests were negative. PEU in one patient showed a high sperm concentration and presented better motility than in the ejaculate [7].

Puri et al. [22] enrolled seven patients for SA, although two of the patients could not collect the sample despite several attempts; however, their PEU was negative for spermaturia. Among the remaining five participants, a high percentage of immotile sperm (70–95%) was observed, along with many leukocytes, within the normal pH range (7.2–8.0). Sperm counts ranged from 24 to 80 million and were below 40 million in two cases. Two patients exhibited prolonged liquefaction times exceeding 30 min; additionally, abnormal viscosity of semen was noted in two cases. Anomalous agglutination of spermatozoa was observed in four cases.

In the experience reported by Caione and Nappo [23], based on 24 patients, normal seminal parameters were reported in 23 cases (95.8%), while 1 patient with CRF and recurrent epididymitis showed azoospermia.

López Pereira et al. [24] enrolled 15 patients, 6 of whom (40%) collected semen samples and PEU. Three out of six (50%) had a semen pH > 8.1. One patient (16.6%) had pH = 8.3, with a below-normal sperm count (4.5 million/mL, total 13.5 million) and reduced motility (25%). Sperm motility was above 32% in five patients (83.3%). Three patients (60%) showed progressive motility close to the 5th percentile (35%). Anomalous sperm forms accounted for 97% in one case (16.6%). In three cases (50%), anomalous forms were close to the upper limit (90–94%). All six patients had normal semen volume, liquefaction time, and viscosity, with negative semen cultures and no IgA antibodies and/or leukocytes. PEU was positive for sperm in all patients; three (60%) exhibited volumes of 0.4 mL, 0.5 mL, and 0.9 mL.

Keihani et al. [9] evaluated five semen samples presenting a normal volume ranging from 1.5 to 5.0 mL. Four out of five samples (80%) exhibited normal pH (>7.2), sperm count, motility, progressive motility, and morphology. One of the patients also presented pyospermia and a history of recurrent epididymal orchitis. One of the five patients (20%) had a slightly lower than normal sperm count, measuring 18 million/mL (reference range: >20 million/mL), associated with low motility, abnormal viscosity, and prolonged liquefaction time.

Finally, in the retrospective study by Çetin et al. [25], SA was available only for three patients: two (66.6%) were infertile (one had oligospermia, the other azoospermia) and they received kidney transplantation; the remaining patient was reported as ‘fertile’ but had not yet achieved conception. This infertility was attributed to a female factor.

The data on seminal parameters described in the different studies are summarized in Table 7.

## 4. Discussion

In most recent studies, PUV patients present fertility rates similar to normal and healthy individuals [2]. Despite the anatomical abnormalities, paternity rates seem to be not significantly different between the two groups [2,5,6].

However, poorer fertility in PUV patients would be expected, considering that 12–16% of them undergo surgery for cryptorchidism, a significant proportion progress to CKD (which can impact fertility), and another portion suffers from recurrent epididymitis [2]. Additionally, urethral dilation can persist after EVR, influencing varying degrees of urinary reflux into the seminal ducts [23].

The analyzed case reports defined the possibility of diagnosing PUVs in adult patients presenting with LUTS, AE, and IF. A form of obstructive RE was described, which in two cases [20,21], was concurrent with inflammation of the accessory seminal glands (i.e., prostatitis or prostate-vesiculitis). Additionally, many patients exhibited ejaculatory disorders, including hypospermia and AE [1,11,12,18,19,20,21], as well as IF [1,11,12,19,20]. This finding suggests a direct association between PUVs and sexual or reproductive disorders. In one case, azoospermia was confirmed through PEU, suggesting obstructive RE [1]. Moreover, EVR appears to be a common therapy for these patients, since it is capable of facilitating conception, sometimes combined with antibiotic therapy in cases of accessory seminal gland infections [20,21]. After surgical treatment, no pathological alterations of semen were reported, when executed [1,12,20,21].

Differently from case reports, the analyzed case series revealed a significantly low rate of long-term follow-up. The high number of patients lost to follow-up or with irretrievable contact information suggests a critical gap in continuous care pathways, underlining potential challenges in tracking and monitoring patients’ long-term health outcomes. The relatively low enrollment rate implies significant difficulties in conducting comprehensive studies among PUV patients. Therefore, the currently described paternity rates in PUV patients could be flawed by attrition bias, potentially not truly representative of the reference population.

Similarly, the low rate of patients who underwent SA let seminal parameters evaluation be considered purely informative, as enrolled participants may not represent the study population. Overestimation or underestimation of PUV effects on fertility cannot be ruled out. Consequently, the currently available data may be less generalizable than previously described [2].

Additionally, the patients’ ages were distributed across a wide range, with some narrower (e.g., 18–35 years) and others broader (e.g., 18–57 years). The lowest average age was 19.6 years, while the highest was 38 years, suggesting a significant variation among the different considered groups. Since age can significantly impact fertility and seminal parameters, even this aspect can represent a confounding bias.

While some studies conducted interviews and assessed seminal parameters [16,18,20,21], others focused only on one of these aspects [8,9,25] or performed a retrospective analysis of clinical data [22]. This variability highlights the lack of uniformity in fertility assessment, and the consequent heterogeneity impedes the results’ comparability. Moreover, these studies did not address any potential additive factor that could further impact fertility [25].

Furthermore, a significant variability in conception and IF rates among PUV patients was evident but, in almost all studies, the data were incomplete. Only one work reported a 100% CR among 11 non-uremic patients who had attempted pregnancies [8]. CR was 15% in Çetin et al.’s experience, although compromised by the lack of clinical data for over half of the patients (53.12%) [25]. Among the remaining studies, four did not enumerate patients who desired conception or attempted to conceive [6,7,23,24], hindering the understanding of real CRs. In one study, none of the patients attempted conception [22], and another work did not assess conceptions [9].

Six studies reported alterations in ejaculatory dynamics. Slow ejaculation ranged from 5% [9] to 38% [7], while AE was 1.49% to 5.97% [6,7], exhibiting some variations attributed to previous surgery consequences, such as BNI [6,9].

SA in PUV patients presented recurring abnormalities, including increased viscosity [7,22], elevated pH [7,24], and significant alterations in motility and progressive motility [7,22,24]. In one study, all the patients exhibited considerable leukocytospermia, despite the absence of suggestive clinical signs for seminal gland infections [22]. In three works PEU revealed sperm cells with higher mobility compared to those present in ejaculate in one case [7], seminal fluid volumes ranging from 0.4 to 0.9 mL in three patients [24], and no sperm in the two subjects who were unable to collect semen [22].

In addition to RE and AE, several pathogenetic mechanisms have been hypothesized to explain infertility and semen alterations in PUV patients. First, PUVs determine a typical dilation of the posterior urethra that allows reflux into the vas deferens, and this condition does not completely regress after EVR, thus affecting the testicular and prostatic components of the semen [23]. Secondly, elevated pH was reported in association with semen viscosity, and both are attributable to increased coagulant factors from the seminal vesicles or decreased liquefaction enzymes produced by the seminal vesicles and prostate, for example, prostate-specific antigen (PSA). Similarly, high pH could result from reduced secretion of prostatic citric acid and acid phosphatase, major contributors to seminal plasma acidity. Elevated pH is notably present in expressed prostatic secretion of patients with prostatitis [7,22]. The presence of subclinical inflammatory conditions is further supported by the finding of leukocytospermia [22], and several cases of PUV patients suffering from recurrent epididymitis have been reported [9,23,26].

Lastly, a single study indicated that CR appears to be influenced more by renal function itself than by the impact of PUVs on the urethral–seminal district [8]. Studies have shown that patients with reduced GFR (e.g., <30 mL/min) are at higher risk of infertility due to renal tubular damage, hormonal dysfunction, and alterations in the vascular system, which can indirectly affect sperm production and sexual function [23,27,28].

In particular, patients with very low GFR (e.g., 25 mL/min) who require dialysis or renal transplantation often experience hormonal and sexual dysfunctions, including erectile difficulties and retrograde ejaculation, which further contribute to reduced fertility [29,30]. Although data on fertility in PUV patients are limited, some studies suggest that preserved renal function (GFR > 60 mL/min) is associated with better fertility outcomes compared to patients with advanced renal insufficiency [31].

Furthermore, it is important to consider that progressive renal damage in PUV patients, who are often diagnosed in childhood, develops over time, and renal function may decline with age, negatively affecting semen quality and the ability to conceive [1,29,32]. Therefore, long-term monitoring of renal function in PUV patients is essential to optimize fertility management.

### 4.1. Limits of the Research

Several limitations of this comprehensive review must be acknowledged. Firstly, the entire structure is built on a body of low-quality studies. The quality of the included works, primarily case reports and small case series, inherently limits the generalizability of the findings. These sources provide valuable insights into a poorly explored field but do not support robust statistical generalizations or causal inferences. Despite these limitations, we included all the available evidence to offer a comprehensive overview and to highlight the critical gaps in the literature, which demand further high-quality research.

Secondly, this work is a narrative review, and as such, it is subject to the inherent limitations of this research methodology, including potential selection bias and the absence of a systematic approach to literature synthesis. Differently from systematic reviews, the lack of a formalized search strategy and the narrative nature of data interpretation reduces the ability to draw robust, evidence-based conclusions.

Thirdly, while this review provides an overview of the topic, it does not address specific management strategies or offer concrete solutions for clinical practice. This absence limits the practical application of the findings in everyday clinical settings. To address this gap, the following section will propose potential strategic solutions based on the available evidence.

Moreover, long-term follow-up data are scarce in the available studies. Many of the reports lack detailed long-term outcomes, particularly regarding fertility and paternity in PUV patients. This makes it difficult to fully assess the enduring impact of posterior urethral valves on reproductive health, limiting the scope of the review.

The heterogeneity of study designs, outcome measures, and follow-up durations complicates the interpretation of findings. However, some consistent patterns, such as the association of PUVs with altered seminal parameters and infertility, suggest the need for standardized assessment protocols. Future research should focus on prospective registries and long-term follow-ups to ensure comprehensive data collection and improve patient management.

### 4.2. Future Perspectives

The current literature does not offer a clear and statistically rigorous view of CRs and seminal parameters of PUV patients. Having highlighted a substantial difficulty in monitoring these patients through cross-sectional and/or retrospective studies, the need to redefine appropriate study protocols emerges, thus advocating for prospective enrollment. Similarly to those with chronic conditions gathering multisystemic effects, PUV patients should be integrated into dedicated care pathways afferent at a multidisciplinary team.

Transitional processes aim to ensure optimal and multidisciplinary care in adulthood, educating patients to assume responsibilities in managing their care pathways, beginning in childhood and ensuring the progressive transition to a dedicated adult team. PUV represents the ideal candidate for transitional processes, encompassing clinical issues in urology, nephrology, psychology, psychiatry, and sometimes endocrinology, alongside potential ongoing nursing support (e.g., intermittent self-catheterization and/or stoma management). The ideal multidisciplinary team would ensure comprehensive care among all the domains contributing to the overall quality of life.

Additionally, the development of a national registry would ensure a proper collection of the various effects of PUV, beyond the traditionally studied domains. Encompassing the renal assessment, national databases would also record surgical interventions, recurrent UTIs, and comorbidities (e.g., obesity, systemic diseases, smoking, and alcoholism) [32]. A standardized approach like this would constantly ensure a clinical evaluation of all the health domains, embracing entirely the psychosocial dynamics. A proper care protocol should achieve a baseline semen evaluation after puberty, incorporating PEU, sperm culture, and prostate-vesicular and epididymal-scrotal ultrasound [33], to early detect inflammatory conditions.

Advanced informatics systems could support these efforts, facilitating the development of remote monitoring platforms, including telemedicine [34]. Moreover, the near future foresees the development of digital twins, avatars remotely representing medical experts in virtual reality. This technology would enable expert care coordination within multidisciplinary teams, even from different geographical locations. Interdisciplinary professionals and patients could meet in virtual rooms within the metaverse for collaborative meetings [35].

Such a proposed pathway, characterized by centralized care, national registries, unified care protocols, and digitalization, would ensure comprehensive and continuous patient management. The real-time monitoring across various health domains of PUV patients would enable proper interventions in areas of need.

## 5. Conclusions

PUVs can contribute to infertility through several mechanisms: persistent posterior urethral dilation allows urinary reflux into the seminal ducts, leading to inflammatory changes; elevated pH levels into the seminal plasma, often associated with chronic prostatitis, impair sperm motility; and CRF has a direct negative impact on spermatogenesis. Addressing these factors requires a multidisciplinary approach that involves urology, nephrology, and reproductive medicine. Fertility and seminal parameters in PUV patients represent an under-investigated area. Improving follow-up could allow for more accurate identification of potential long-term effects of PUVs on male fertility and could lead to targeted interventions to improve the related outcomes. The development of shared protocols and international databases, along with healthcare digitization, could enhance the clinical management of PUVs.

## Figures and Tables

**Table 1:** Search summary.

Items	Specification
Search date	April 2024
Databases	PubMed, Cochrane Database, Scopus and Embase
Search terms (including MeSH ed Boolean operators)	(posterior urethral valve) AND ((fertility) OR (ejaculation) OR (sexual dysfunction) OR (potency) OR (pregnancy rate) OR (sexual function) OR (paternity))
Timeframe	No temporal limits
Exclusion criteria	Editorials, commentaries, abstracts, reviews, and book chapters
Selection process	M.D.C. and S.G.N.
Further considerations, if applicable	No further considerations

**Table 2:** Glossary of main key terms, according to WHO.

Key Term	Definition
Infertility	The inability to conceive after one year of regular, unprotected sexual intercourse.
Semen Analysis	A laboratory test used to evaluate sperm count, motility, morphology, vitality, and other characteristics of semen to assess male fertility.
Retrograde Ejaculation	A condition in which semen, instead of being ejaculated out of the penis, flows backward into the bladder during orgasm. This can affect fertility because the sperm does not exit the body and cannot reach the egg for fertilization.
Sperm Count	The total number of sperm cells present in a semen sample. A normal sperm count typically ranges from 15 million to over 200 million sperm per milliliter of semen. A low sperm count (oligospermia) can reduce the chances of successful fertilization.
Semen Volume	The amount of fluid produced during ejaculation. It typically ranges from 2 to 5 milliliters. Low semen volume (hypospermia) can affect fertility by reducing the number of sperm available to reach and fertilize the egg.
Sperm Concentration	The number of sperm per milliliter of semen. Normal sperm concentration is considered to be at least 15 million sperm per milliliter.
Sperm Motility	The percentage of sperm cells that actively move. A normal motility rate is greater than 40%, with at least 32% of sperm showing progressive motility (moving forward in a straight line).
Sperm Morphology	The shape of sperm cells. Normal sperm cells have an oval head with a well-defined acrosome (the part of the sperm that helps it penetrate the egg). A normal morphology rate is typically greater than 4–14% of sperm with normal shape.
Sperm Vitality	The percentage of living sperm cells in the sample. A normal vitality rate is greater than 58% of live sperm cells.

**Table 3:** Summary of included studies in chronological order and categorized by type.

#	Authors	Year	Type of Study
**1**	Waligora [19]	1980	Case report
**2**	Hama et al. [18]	1981	Case report
**3**	Pàramo et al. [20]	1983	Case report
**4**	Woodhouse et al. [7]	1989	Cross-sectional
**5**	Dutkiewicz [21]	1994	Case report
**6**	Puri et al. [22]	2002	Cross-sectional
**7**	Holmdahl e Sillén [8]	2005	Cross-sectional
**8**	Caione e Nappo [23]	2011	Cross-sectional
**9**	Taskinen et al. [6]	2012	Cross-sectional
**10**	López Pereira et al. [24]	2011	Cross-sectional
**11**	Agbugui e Omokhudu [12]	2014	Case report
**12**	Keihani et al. [9]	2016	Cross-sectional
**13**	Mbaeri et al. [11]	2020	Case report
**14**	Rajih et al. [1]	2020	Case report
**15**	Çetin et al. [25]	2020	Retrospective

**Table 4:** Assessment of study quality according to Murad et al.’s tool [16].

#	Authors		Domains		
		Selection	Ascertainment		Reporting
**1**	Waligora [19]	1	1	1	1
**2**	Hama et al. [18]	1	1	0	1
**3**	Pàramo et al. [20]	1	1	1	1
**4**	Woodhouse et al. [7]	1	1	1	1
**5**	Dutkiewicz [21]	1	1	1	1
**6**	Puri et al. [22]	1	1	1	1
**7**	Holmdahl et Sillén [8]	1	1	1	1
**8**	Caione et Nappo [23]	1	1	1	1
**9**	Taskinen et al. [6]	1	1	0	1
**10**	López Pereira et al. [24]	1	1	1	1
**11**	Agbugui et Omokhudu [12]	1	1	1	1
**12**	Keihani et al. [9]	1	1	0	1
**13**	Mbaeri et al. [11]	1	1	1	1
**14**	Rajih et al. [1]	1	1	1	1
**15**	Çetin et al. [25]	1	1	0	1

**Table 5:** Summary of selected case reports. * Hama et al. [18] considered the restoration of antegrade ejaculation as the sole outcome. Legenda: NR = not reported; AE = anejaculation; PEU = post-ejaculation urine; TSC = total sperm count; IF = infertility; RE = retrograde ejaculation; EVR = endoscopic valve resection; ATB = antibiotics; WBC = white blood cells; HPF = high power field; NV = normal value; HYS = hypospermia; + = conception was achieved.

#	Age	Diagnosis	Baseline Sperm Parameters	Pathogenetic Mechanism	Treatment	Outcomes	
						Conception	Seminal Parameters
**1 [19]**	ND	AE + IF	NR	RE	EVR	+	NR
**2 [18]**	23	AE				NR *	NR
**3 [20]**	30	AE + IF	PEU: pH 6.5, *E. coli* bacteriuria; TSC 180 × 10^6^ with low motility (immotile sperms 55%; grade 3 movement 5%), normal morphology, fructose absent	RE + chronic prostatitis + bladder diverticulum	EVR + ATB + diverticulectomy	+	TSC 51 × 10^6^/mL (178.5 × 10^6^/ejaculation), volume 3.5 mL, motility 70%. Normal morphology and fructose. Seminal plasma alterations: (4–8 WBC/HPF; citric acid 122 mg/mL (NV > 400); zinc 31 pg/mL (NV 238 ± 89), negative culture
**5 [21]**	20	AE	PEU: TSC 15–30 immotile/HPF, normal morphology, 3–5 WBC, negative culture	RE + chronic prostatitis	EVR + ATB	NR	TSC 81 × 10^6^/mL, active motility 80%, 4–6 WBC/HPF
**11 [12]**	40	AE + IF	NR	RE	EVR	+	TSC 38 × 10^6^, volume 3.5 mL, motility 55%, normal forms 40%
**13 [11]**	34	AE + IF	NR	RE		+	NR
**14 [1]**	45	HYS + IF	HYS (volume 1 mL) ed azoospermia. PEU: azoospermia	RE		+	TSC 22 × 10^7^/mL, volume 2 mL, normal forms 41%, progressive motility 40%

**Table 6:** Summary of the case series. Legenda: NR = not reported; SA = semen analysis; INT = interview; HYS = hypospermia; AE = anejaculation; C = conceptions; IF = infertility; EVR = endoscopic valve resection; BNI = bladder neck incision; NA = no attempts; * = not specified if subjects were actively seeking pregnancy.

#	VUP Patients [N]	Patients Enrolled [E] (% N)	Age	Previous Treatment	Assessment	Semen Analysis (%E; %N)	Clinics (% E)	Conceptions (% E) and Infertility
**4 [7]**	105	21 (20%)	Range 19–37 Mean 24.6	NR	SA + INT	9 (42.85%; 8%)	8 (38%) HYS 1 (4.7%) AE	C = 3 (14%); IF = 3 (14.2%) *
**6 [22]**	156	8 (5.12%)	Range 16–21 Mean 17.5	EVR	SA + INT	7 (77.8%; 4.5%)	Healthy	NA
**7 [8]**	27	19 (70%)	Range 31–44 Median 36	14/27 (52%) BNI; 2/27 (7%) EVR; perineal urethrotomy (% NR)	INT	0 (0%)	6 (31.5%) uremic: 13 (68.5%) no-uremic	C = 11 (85%) of non-uremic; 2 (10.5%) of non-uremic NA; no patients of 6 (31.5%) with uremia had children
**8 [23]**	45	24 (53.3%)	Range 18–34 Mean 23	EVR	SA + INT	24 (100%; 53.3%)	1 (4.16%) recurrent epididymitis; 2 (8.33%) HYS	C = 0% *
**9 [6]**	200	67 (33.5%)	Range 18–57 Mean 38	EVR ± BNI	INT	0 (0%)	4 (5.97%)	C = 33 (49.25%); IF = 8 (12%) *
**10 [24]**	47	15 (31.9%)	Range 18–35 Mean 24	NR	SA + INT	6 (12.7%)	1 (6%) HYS	C = 1 (7%) *
**12 [9]**	541	18 (3.32%)	Range 18–29 Mean 21.2	BNI	SA + INT	5 (0.9%)	1 (5%) HYS	NR
**15 [25]**	89	32 (35.9%)	Range 18–46 Median 26	NR	Retrospective Analysis of clinical data	3 (3.37%)	4 (12.5%) HYS	C = 5 (15%); 8 (25%) desiring offspring *, IF = 2 (6.25%); 17 (53.12%) missing data

**Table 7:** Summary of the seminal parameters reported in the different analyzed case series. Legenda: SA = semen analysis; TSC = total sperm count; PEU = post-ejaculation urine; WBC = white blood cells; NV = normal value; IF = infertility.

#	Samples Analyzed	Characteristics
**4 [7]**	9	SA: 9 (100%) normal values, 5 (56%) increased viscosity and pH (>8.0). “Shaking movements” as for antibodies component, agglutination negative. PEU executed on 7 patients: 6 had normal TSC, no differences in sperm volume and TSC between patients with and without slow ejaculation, and 1 had higher TSC and higher mobility than SA.
**6 [22]**	5	SA: 2/5 (40%) high liquefaction time. 5/5 pH 7.2–8. TSC: 24–80 milioni per campione. No oligospermia. 70–95% immotility. 100% leucokytospermia. 4/5 (80%) atypical agglutination. 2/5 (40%) augmented viscosity.
**8 [23]**	24	SA: 1/24 (4.2%) azoospermia (recurrent epididymitis). 23/24 (95.8%) normal seminal values.
**10 [24]**	6	SA: 6/6 (100%) normal volume, liquefaction time and viscosity, no IgA antibodies, no WBC, negative culture. 3/6 (50%) pH > 8.1. 1/6 (16.6%%) pH = 8.3 e (4.5 × 10^6^/mL, total 13.5 × 10^6^/mL) e reduced motility (25%). 5/6 (83.3%) motility > 32%, progressive motility close to 5° centile in 3/5 (35%). Anomalous forms: 97% in 1/6 (16.6%), close to upper limit (90–94%) in 3/6 patients (50%). PEU: 6/6 (100%) spermaturia. 3/5 sperm volume of 0.4 mL, 0.5 mL and 0.9 mL.
**12 [9]**	5	SA: 5/5 (100%) normal volume (1.5–5.0 mL). 4/5 (80%) normal pH (>7.2), TSC, motility, progression, and morphology. One of them had pyospermia and recurrent orchi-epididymitis. 1/5 (20%) TSC 18 × 10^6^/mL (NV: > 20 × 10^6^/mL), low motility, abnormal viscosity, and liquefaction time.
**15 [25]**	3	SA: 2/3 (66%) IF kidney transplanted: 1 oligospermia and 1 azoospermia. 1/3 (33%) fertile.

## Data Availability

Not applicable. No new data have been generated.

## Abbreviations

AE = anejaculation; ATB = antibiotics; BNI = bladder neck incision; C = conceptions; CR = conception rate; CRF = chronic renal failure; ED = erectile dysfunction; EVR = endoscopic valve resection; HPF = high power field; HYS = hypospermia; IF = infertility; INT = interview; LUTS = lower urinary tract symptoms; NA = no attempts; NR = not reported; NV = normal value; PEO = population-exposure-outcome; PEU = post-ejaculation urine; PUV = posterior urethral valve; RE = retrograde ejaculation; SA = semen analysis; SANRA (scale) = Scale for the Assessment of Narrative Review Articles; TSC = total sperm count; UTI = urinary tract infection; US = ultrasonography; WBC = white blood cells, WHO = World Health Organization.
